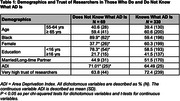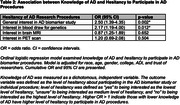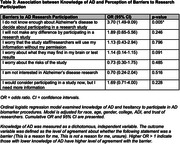# Impact of Knowledge About Alzheimer’s Disease on Interest in Participation in Biomarker Research

**DOI:** 10.1002/alz.092946

**Published:** 2025-01-09

**Authors:** Taylor Etchison, Johanne Eliacin, Angelina J Polsinelli, Ralph Richards, Mollie Richards, Christopher Campbell, Pamella Shaw, Sujuan Gao, Sarah Van Heiden, Shannon L. Risacher, Hugh C Hendrie, Andrew J. Saykin, Sophia Wang

**Affiliations:** ^1^ Indiana University School of Medicine, Indianapolis, IN USA; ^2^ Indiana Alzheimer’s Disease Research Center, Indianapolis, IN USA; ^3^ VA HSR&D Center for Health Information and Communication, Indianapolis, IN USA; ^4^ Regenstrief Institute Inc., Indianapolis, IN USA

## Abstract

**Background:**

Previous studies suggest limited knowledge about Alzheimer’s Disease (AD) is a barrier to underrepresented group participation in AD research. Connections between knowledge of AD and factors like social determinants of health or confidence in biomarker research have not been carefully examined. We hypothesized perceived knowledge about AD would be associated with research hesitancy independent of sociodemographics and trust of researchers.

**Method:**

The AD‐REACH study surveyed 399 research‐naïve non‐Hispanic Black and white adults 55 years and older living in Indianapolis, Indiana. Participants reported perceived knowledge of AD on a 5‐point scale (5 = “I know what [AD] is, what causes it, and how to manage and prevent it;” 1 = “I know nothing at all”). Knowledge was dichotomized into higher (≥ 3) and lower (< 3) levels. Demographics were compared using chi‐squared tests and t‐tests. Ordinal logistic regression models examined the association between perceived knowledge about AD and outcome variables (e.g., hesitancy towards research participation) and were adjusted for sociodemographics and very high trust of researchers.

**Result:**

Those who reported being Black, male, from a higher Area Deprivation Index, or having less than 16 years of education had lower perceived knowledge about AD (**Table 1**). Trust of researchers was not associated with perceived knowledge. Those reporting lower AD knowledge described more hesitancy to participate in biomarker research (53.0% vs 72.7%, OR 2.50, 95% CI 4.35‐1.39, p = 0.002) and blood draw (62.3% vs 80.9%, OR 2.17, 95% CI 4.00‐1.19, p = 0.012) but not neuroimaging procedures (**Table 2**). Those with lower perceived knowledge of AD were more likely to need additional information to make decision about whether to participate in research (58.1% vs 29.9%, OR 3.70, 95% CI 9.09‐1.49, p = 0.005) (**Table 3**).

**Conclusion:**

Populations with health disparities report lower knowledge of AD, and lower perceived knowledge is associated with research hesitancy, especially for biomarker procedures. Perceived AD knowledge is also independent from trust, suggesting the feasibility of a two‐pronged intervention to foster diverse participation in AD biomarker research. Future studies will need to confirm these findings in other cohorts and examine how culturally tailored strategies to increase AD knowledge may reduce research hesitancy among underrepresented groups.